# An exploratory metabolomic comparison of participants with fast or absent functional progression from 2CARE, a randomized, double-blind clinical trial in Huntington’s disease

**DOI:** 10.1038/s41598-023-50553-y

**Published:** 2024-01-11

**Authors:** Andrew McGarry, Krystal Hunter, John Gaughan, Peggy Auinger, Thomas N. Ferraro, Basant Pradhan, Luigi Ferrucci, Josephine M. Egan, Ruin Moaddel

**Affiliations:** 1 Department of Neurology, Cooper University Hospital and Cooper Medical School at Rowan University, Camden, NJ USA; 2 Department of Medicine, Cooper Medical School at Rowan University, Camden, NJ USA; 3 Department of Neurology, Center for Health and Technology, University of Rochester, Rochester, NY USA; 4 Department of Biomedical Sciences, Cooper Medical School at Rowan University, Camden, NJ USA; 5 Department of Psychiatry, Cooper Medical School at Rowan University, Camden, NJ USA; 6 Biomedical Research Center, National Institute on Aging, National Institutes of Health, Baltimore, MD 21224 USA

**Keywords:** Neuroscience, Systems biology, Biomarkers, Diseases, Medical research

## Abstract

Huntington’s disease (HD) is increasingly recognized for diverse pathology outside of the nervous system. To describe the biology of HD in relation to functional progression, we previously analyzed the plasma and CSF metabolome in a cross-sectional study of participants who had various degrees of functional impairment. Here, we carried out an exploratory study in plasma from HD individuals over a 3-year time frame to assess whether differences exist between those with fast or absent clinical progression. There were more differences in circulating metabolite levels for fast progressors compared to absent progressors (111 vs 20, nominal p < 0.05). All metabolite changes in faster progressors were decreases, whereas some metabolite concentrations increased in absent progressors. Many of the metabolite levels that decreased in the fast progressors were higher at Screening compared to absent progressors but ended up lower by Year 3. Changes in faster progression suggest greater oxidative stress and inflammation (kynurenine, diacylglycerides, cysteine), disturbances in nitric oxide and urea metabolism (arginine, citrulline, ornithine, GABR), lower polyamines (putrescine and spermine), elevated glucose, and deficient AMPK signaling. Metabolomic differences between fast and absent progressors suggest the possibility of predicting functional decline in HD, and possibly delaying it with interventions to augment arginine, polyamines, and glucose regulation.

## Introduction

Huntington’s disease (HD), a fatal neurodegenerative disorder characterized by progressive functional decline, is increasingly recognized for diverse pathology outside of the nervous system^1–6^. Mutant huntingtin protein is ubiquitously expressed throughout the body, an observation consistent with data suggesting that other tissues or cell types experience vulnerability and dysfunction. This growing appreciation further motivates a better understanding of the biology underpinning these abnormalities, which may offer new opportunities for disease intervention.

We recently analyzed the plasma and CSF metabolome in a cross-sectional study of participants with HD of varying disease severity as measured by the Total Functional Capacity (TFC) score, an accepted measure of disease progression^7,8^. We found changes in the urea cycle, arginine (Arg), citrulline (Cit), glycine (Gly), serine (Ser), cholesterol esters (CEs), diacylglycerides (DGs), triacylglycerides (TGs), phosphatidylcholines (PCs), phosphatidylethanolamines, and sphingomyelins (SMs) correlated to disease progression^7^. Here, we sought to examine whether these observations could be substantiated by looking in plasma from individuals over time, and also whether differences might exist between those with faster or slower clinical progression. To explore these possibilities, we analyzed plasma from participants receiving placebo in 2CARE, a 5-year, randomized, double-blind clinical trial of Coenzyme Q10 in HD^9^.

## Results

After selection, seven 2CARE participants with fast progression (FP) from Screening to Year 3 and 13 participants with absent progression (AP) over the same interval were analyzed. Demographics, concomitant medications, number of samples used per timepoint, and longitudinal TFC values for these participants are shown in Table 1. The mean baseline screening TFC value in the FP group was 11.1 (range 9–13) and 10.9 in the AP group (range 9–13) (Table 1). Ages of participants ranged from 24 to 67 years, with a mean age of 44 years for FP and 41 years for AP. Nine participants were female: 3/7 in FP, and 6/14 in AP. Mean CAG repeat length in FP group was 50 (range 40–66), with two notable expansions of 63 and 66 repeats, whereas the AP group averaged 44 repeats (range 41–49). All participants contributed plasma at Screening. In FP, three contributed at Year 3 (01F, 05F, 06F); ages were 23, 48, and 67 years, with CAG repeats of 63, 45 and 40 respectively. In AP, ten contributed at Year 3 (01A–09A, 12A); ages ranged from 25 to 61, with CAGs of 41–49.

**Table 1:** TFC scores, demographics, and concomitant medications for analyzed 2CARE participants.

Fast progression											
Plasma sample N	7	3	5	3	Age	Sex	CAG length	Concomitant medications at screening	Concomitant medications at Year 3	Medical history at screening	Medical history at Year 3
TFC scores	Year										
	Screen	Y1	Y2	Y3							
***01F***	***13***	11	9	***7***	67	M	40/18	Diltiazem Valsartan Nitrofurantoin Pravastatin Insulin Aspart Escitalopram	Diltiazem Valsartan Pravastatin Insulin Aspart Silodosin Buproprion Sertraline	Type I diabetes Diabetic retinopathy Hypercholesterolemia Depression	Type I diabetes Diabetic retinopathy Hypercholesterolemia Depression
02F	9	9	5	N/A	24	F	66/15	Risperidone Baclofen	Atropine ophthalmic	Depression	Depression
03F	13	9	7	6	55	M	43/18	Atorvastatin Botulinum Toxin Escitalopram	Aripiprazole Risperidone Venlafaxine Simvastatin	Hypercholesterolemia Headaches Depression	Hypercholesterolemia Headaches Depression
04F	9	5	1	0	47	M	46/17	Haloperidol Multivitamin Fish Oil Risperidone	Haloperidol Olanzapine Loperamide Hydrocortisone Triamcinolone Omeprazole	Acne Hypercholesterolemia	Acne Hypercholesterolemia Gastric reflux
***05F***	***11***	9	8	***2***	48	F	45/17	Desloratadine Tetrabenazine Duloxetine Temazepam	Wellbutrin Mirtazapine Zopiclone Olanzapine Tetrabenazine	Seasonal allergies Depression Insomnia	Depression Insomnia
***06F***	***12***	11	7	***7***	23	M	63/19	None	Baclofen	Constipation Maxillary cysts	Constipation Maxillary cysts
07F	11	5	5	N/A	41	F	47/17	Budesonide Venlafaxine	Budesonide Venlafaxine Olanzapine	Sarcoidosis (in remission) Depression Allergic rhinitis	Sarcoidosis (in remission) Depression Allergic rhinitis

Absent progression											
Plasma sample N	13	9	12	10	Age	Sex	CAG length	Concomitant medications at screening	Concomitant medications At Year 3	Medical history at screening	Medical history at Year 3
TFC scores	Year										
	Screen	Y1	Y2	Y3							
**01A**	***13***	13	13	***13***	61	M	41/17	Carvedilol Lisinopril Aspirin Fish Oil Lecithin Calcium D Citalopram Buspirone	Aspirin Calcium Citalopram Lecithin Buspirone Carvedilol Lisinopril	Tinnitus Cardiomyopathy Hypertension Irritable Bowel Syndrome Depression Obsessive–compulsive disorder	Tinnitus Cardiomyopathy Hypertension Irritable Bowel Syndrome Depression Obsessive–compulsive disorder
**02A**	***13***	13	13	***13***	43	M	45/22	None	None	None	None
**03A**	***10***	10	10	***10***	47	M	42/23	Hydrocodone/Acetaminophen Loratadine Multivitamin Aspirin B12	Hydrocodone/Acetaminophen Loratadine Multivitamin Aspirin B12 Methylphenidate Nebivolol Pramipexole Citalopram Diphenhydramine	Hypercholesterolemia Back pain Headaches Depression Allergic rhinitis	Hypercholesterolemia Hypertension Back pain Headaches Depression Allergic rhinitis
**04A**	***13***	13	13	***13***	49	M	42/21	Atorvastatin Amlodipine Candesartan Indomethacin	Atorvastatin Amlodipine Candesartan Indomethacin	Hypertension hypercholesterolemia	Hypertension hypercholesterolemia
**05A**	***13***	13	13	***13***	25	F	49/17	Budesonide Terbutaline Multivitamin Betamethasone Medroxyprogesterone	Calcium Vitamin D Ferrous Fumarate Medroxyprogesterone Budesonide Betamethasone Terbutaline	Ovarian cysts Fatigue	Ovarian cysts Fatigue Iron deficiency Asthma
**06A**	***9***	9	9	***9***	40	M	47/22	Fexofenadine Mometasone Omeprazole Paroxetine	Fexofenadine Mometasone Fluoxetine Pantoprazole Alprazolam Quetiapine	Gastric reflux Headaches Irritability Seasonal allergies	Gastric reflux Headaches Irritability Seasonal allergies
**07A**	***11***	11	11	***11***	49	F	42/17	Omega 3 Fish Oil Lorazepam	Fish Oil Lorazepam	Eczema Nephrolithiasis Depression Insomnia Uterine hemorrhage	Eczema Nephrolithiasis Depression Insomnia Uterine hemorrhage
**08A**	***11***	11	11	***11***	37	F	46/17	Dimenhydrinate Tetracycline Folic Acid Venlafaxine Quetiapine Pantoprazole Lamotrigine Axert Simethicone Trazodone.HCl	Dimenhydrinate Folic Acid Pantoprazole Lamotrigine Almotriptan Simethicone Trazodone Venlafaxine Methocarbamol with Paracetamol Terazosin Quetiapine	Acne Iron deficiency anemia Carpal tunnel syndrome Back pain Migraine headaches Bipolar disorder Depression Gastric reflux	Acne Iron deficiency anemia Carpal tunnel syndrome Back pain Migraine headaches Bipolar disorder Depression Gastric reflux
**09A**	***13***	13	13	***13***	41	F	43/21	Ibuprofen	Bupropion Simethicone	Back pain	Depression Back pain
10A	13	13	13	13	32	F	44/23	Clonazepam Drospirenone Hyoscyamine Loperamide Trazodone	Clonazepam Drospirenone Hyoscyamine Trazodone Diclofenac Diphenoxylate with atropine sulfate Citalopram	Irritable bowel syndrome Restless leg syndrome Depression	Irritable bowel syndrome Restless leg syndrome Depression
11A	12	12	12	12	62	F	41/24	Esomeprazole Conjugated estrogens/ medroxyprogesterone Acetaminophen Montelukast Celebrex Olopatadine Erythromycin Escitalopram Haloperidol Docusate Sodium Ranitidine Venlafaxine Fexofenadine Levothyroxine	Esomeprazole Lisinopril Benzonatate Montelukast Erythromycin Escitalopram Haloperidol Docusate Sodium Ranitidine Venlafaxine Fexofenadine Levothyroxine Valproate Hydrocodone/ Acetaminophen	Hypercholesterolemia Gastric reflux Hypothyroidism Depression Osteoarthritis Asthma Ovarian cysts Menorrhagia Headaches	Hypercholesterolemia Gastric reflux Hypothyroidism Depression Osteoarthritis Asthma Ovarian cysts Menorrhagia Headaches
**12A**	**11**	11	11	***11***	46	M	43/17	Sildenafil Multivitamin Vitamin D	Sildenafil Multivitamin Trazodone	Depression Insomnia Erectile dysfunction	Depression Insomnia Erectile dysfunction
13A	10	10	10	*10*	46	M	42/21	Simethicone Fluoxetine Fish Oil Creatine Mometasone Furoate	Simethicone Mometasone Fish Oil Fluoxetine	Actinic keratosis Eczema Gastric reflux Depression insomnia	Actinic keratosis Eczema Gastric reflux Depression insomnia

All contributed plasma at Screening; those contributing plasma at Screening and Year 3 are indicated in bold.

At Screening in the FP group, 4/7 were on antidepressants or anxiolytics, 2/7 on antipsychotics, and one person on tetrabenazine; by Year 3, these were 4/7, 4/7 and 1/7, respectively. For AP at Screening, 6/13 were on antidepressants or anxiolytics, 2/13 on antipsychotics, and none on tetrabenazine; by Year 3, these figures were 9/13, 3/13, and none, respectively. In FP contributing plasma at Year 3, 2/3 were on antidepressants, with one of these also on an antipsychotic and tetrabenazine. For AP plasma analyzed at Year 3, 6/10 were on antidepressants and 2/10 on antipsychotics.

### Pooled analysis 

#### Metabolites with nominally significant differences (p<0.05) and trends (0.05<p<0.10) between screening and Year 3 within the FP and AP groups

Metabolites that were nominally different are reported in Table 2 with circulating concentrations reported in Table S1. In the FP group, 7 participants contributed plasma at Screening, while 3 were available by Year 3. 111 metabolites were nominally different with all decreasing in concentration between Screening and Year 3 (Table 2A), including acyl carnitines, PCs acyl acyl (aa) and acyl alkyl (ae), lysophosphatidyl cholines (LPCs), SMs, CEs, ceramides (Cer), hexosyl ceramides (HexCer), amino acids and biogenic amines. The directional associations of PCs, SMs, Cer and HexCer with TFC were similar to previous observations in plasma^7^. The indoleacetic acid (3-IAA) level had a larger decline by Year 3 in the FP compared to the AP (68.8% v 14.7%). For summated variables, 19 were nominally different and 8 were trending, all reflecting decreases in concentration by Year 3 (Table 2A).

**Table 2:**
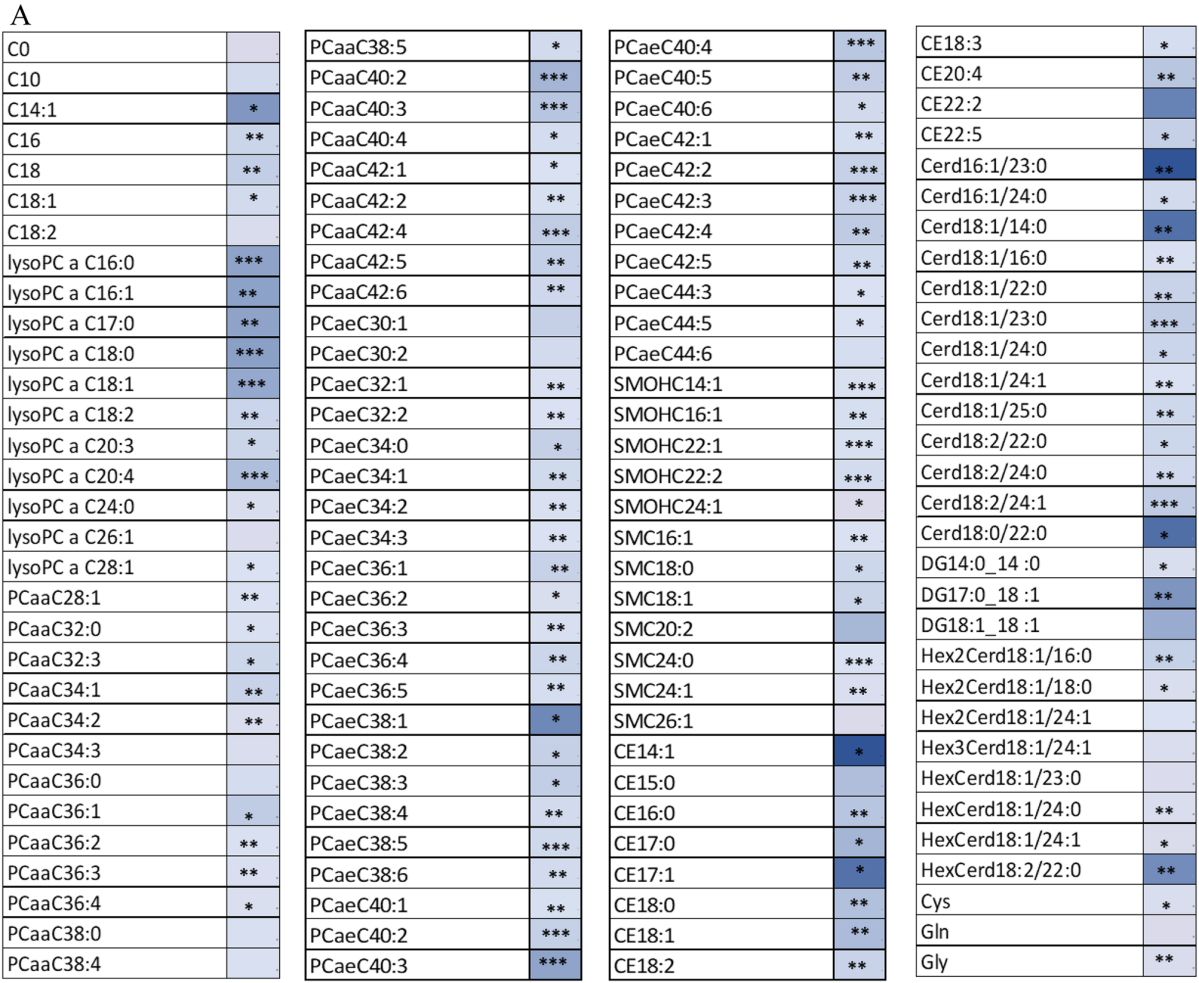

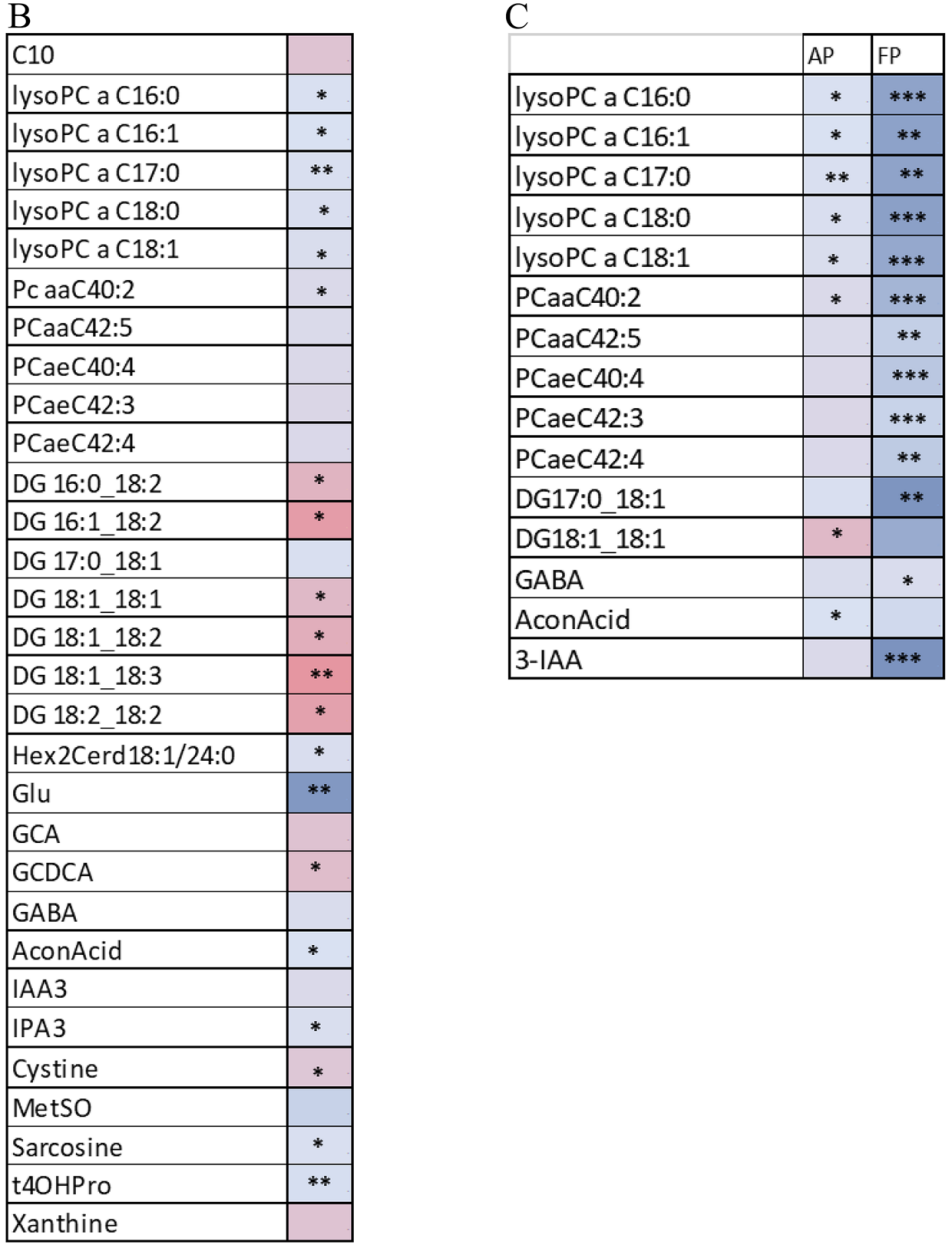
Metabolites that were nominally different (nominal p < 0.05) between Screening and Year 3 in Fast Progressors (A) (n = 7, 3, respectively), Absent Progressors (B) (n = 13, 10 respectively), and both groups (C).

*Indicate nominal significance: *0.01 < p < 0.05; **0.001 < p < 0.01 and ***p < 0.001. No asterisk indicates  trending (0.05 < p < 0.10). Color indicates relative change from Screening to Year 3 (Red = increase; Blue = decrease). Shorthand notation^10^ for the lipids are provided in Table S2.

In the AP group, 13 participants contributed plasma from the Screening visit while 10 contributed at Year 3. 20 metabolites were nominally different (eight increasing and 12 decreasing) between Screening and Year 3 and 11 were trending (Table 2B), including acyl carnitine C10, LPCs, PCs, DGs, Hex Cer, amino acids, bile acids, indoles and biogenic amines. In our previous study DGs decreased with functional progression^7^, while in this study the majority of the DGs associated with AP increased, with only DG17:0/18:1 decreasing. Among sums and ratios, only LPC/PC (decrease by Year 3) and Arg/Ornithine (Arg/Orn) (increase at Year 3) were nominally significant, while cystine/Cys (increase at Year 3) and Serine/Glycine (Ser/Gly) (decrease at Year 3) were trending.

Metabolites that decreased longitudinally in both groups with nominal significance included 5 LPCs and PC aa C40:2 (Table 2C). Others that show nominal significance in one group and trending in the other included DG 17:0/18:1, DG 18:1/18:1, 5 LPCs, GABA, 3-IAA and aconitic acid. C10 trended towards nominal significance in both groups but increased in AP while decreasing in FP. DG 18:1/18:1 increased by Year 3 in AP but was lower at that timepoint in FP groups, while DG 17:0/18:1 decreased over time in both groups.

#### Metabolites with nominally significant differences and trends between FP and AP groups at Screening

At Screening, 33 metabolites were nominally different between FP and AP groups (Table 3A). 30 of these had higher concentrations at Screening in the FP group with the exception of PCaa C32:2, putrescine and spermine, which were higher in the AP group. Metabolites with higher concentrations in the FP group included 7 LPCs, 5 CEs and 6 SMs, Arg, Gly, His, glutamine (Gln), Ser, Trp, betaine, Cit, Orn, glucose, and p-cresol sulfate. Concentrations of Arg, Gln, Gly, His and Orn were also higher at Year 3 in FP than the screening values of these metabolites in the AP group. EPA was present in ~ twofold higher concentration at Screening in the FP group and lower at Year 3, with a trending increase over time in AP. Trp and Kyn levels were higher at Screening in FP, but by Year 3 fell below both Screening and Year 3 values in AP. Plasma glucose levels were ~ 50% higher in FP at Screening but remained stable thereafter in both groups. Total Ser levels were nominally higher at Screening in the FP group and remained numerically higher than the AP group at Year 3; however, concentrations declined by 25% at Year 3 in the FP group (mean 136.66 to 102.23) while remaining relatively more stable in the AP group (97.5 to 90.42), consistent with previous studies^7^.

**Table 3:**
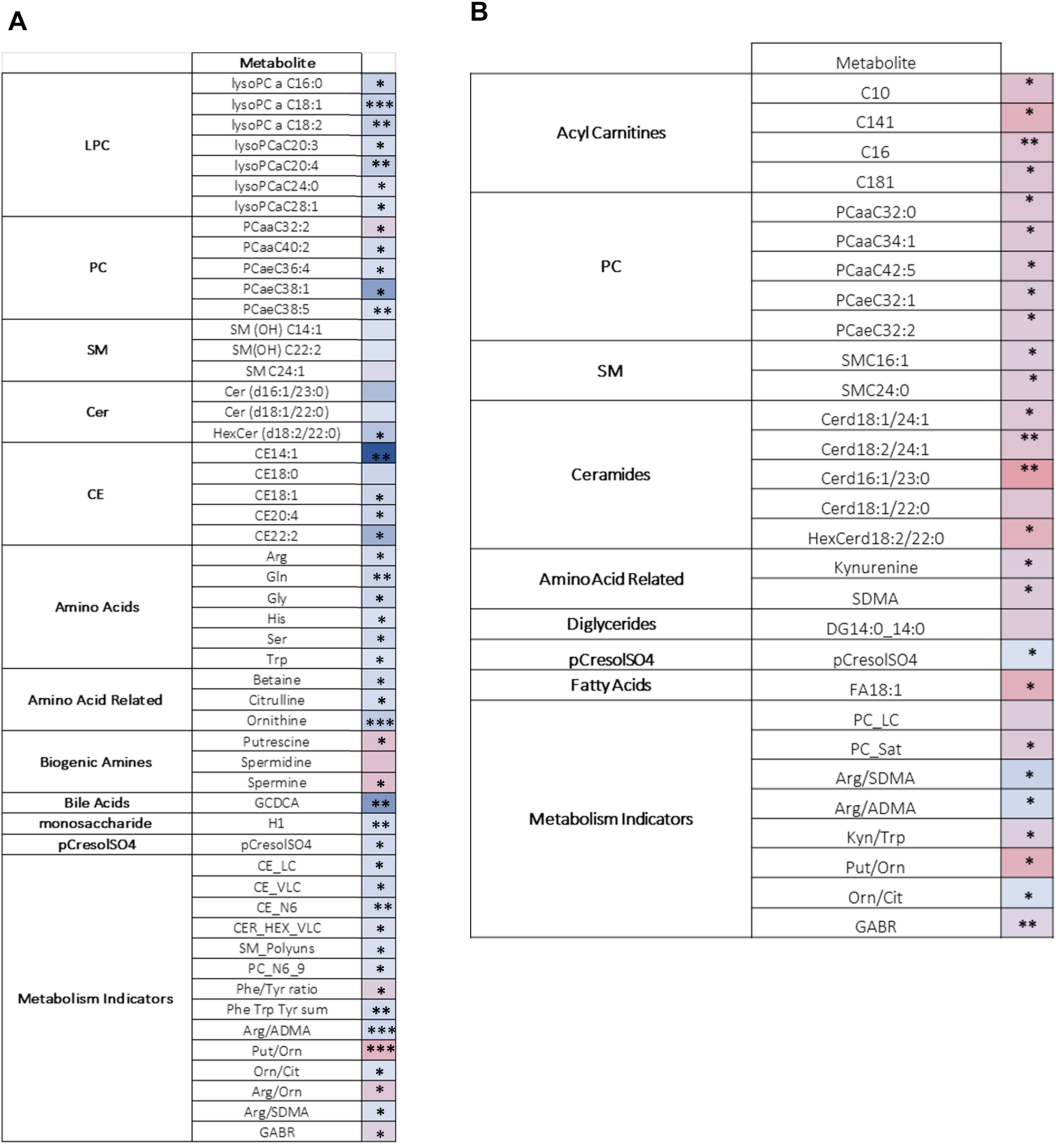
Metabolites with nominally significant differences and trends between FP and AP groups at Screening (A) (n = 7, 13, respectively) and at Year 3 (B) (n = 3, 10, respectively).

*Indicate nominal significance: *0.01 < p < 0.05; **0.001 < p < 0.01 and ***p < 0.001. No asterisk indicates  trending (0.05 < p < 0.10). Color indicates relative difference between Absent Progressors and Fast Progressors (Red = higher in Absent Progressors; Blue = lower in Absent Progressors). Shorthand notation^10^ for the lipids are provided in Table S2.

Seven ratios were nominally significant between FP and AP at Screening: Arg/Orn, Arg/ADMA, Arg/SDMA, Putrescine/Orn, Phe/Tyr, and global arginine bioavailability ratio (GABR (Arg/Orn + Cit)). Seven sums of related metabolites are nominally significant: aromatic amino acids, LCFA CEs, VLCFA CEs, Ω -6 fatty acids, very long chain (VLC) HexCer, SMs and polyunsaturated fatty acids, and the sum of PCs, Ω-6, and Ω-9 fatty acids.

#### Metabolites with nominally significant differences and trends between FP and AP groups at Year 3

At Year 3, 20 metabolites were nominally different between FP and AP groups (Table 3B). These included acylcarnitines, PCs, SMs, Cer, DG, Kyn and SDMA and fatty acid (FA) 18:1, all lower in FP. Concentrations were numerically higher at Screening in the FP group for all but two (PC aa C42:5, DG 14:0/14:0), with AP group metabolites remaining more stable through time.

Six ratios were nominally significant between FP and AP: Arg/SDMA, Arg/ADMA, Kyn/Trp, Putrescine/Orn, Orn/Cit, and GABR. The Arg/SDMA and Arg/ADMA ratio values were significantly higher in the FP group at Year 3. Sums of PC + LC and saturated PCs were also nominally different at Year 3, with higher total concentrations in the AP group.

#### Metabolites with nominally significant differences and trends between FP and AP groups at both baseline and Year 3

Markers that were nominally different between the groups at Screening and Year 3 were HexCer (d18:2/22:0) and the sum of hexoses (H1) (Table 3A,B). Markers trending at both timepoints in both FP and AP groups were SM (OH) C22:2, SM C24:1, Cerd18:1/22:0, and Cerd16:1/23:0. These followed the same pattern of higher starting concentrations and steeper decline in FP compared to the relative stability in the AP group. The exception was glucose, which was higher in the FP group at Screening and remained higher at Year 3.

Three ratios are nominally different between FP and AP at both Screening and Year 3: Arg/ADMA, Putrescine/Orn, and GABR.Arg/ADMA was higher at Screening and Year 3 for FP, while Putrescine/Orn and GABR were higher at Screening and Year 3 in AP.

## Discussion

The current study offers a unique opportunity to examine the relationship between rates of functional progression and changes in the plasma metabolome for placebo participants from 2CARE, a large, 5-year interventional clinical trial in HD. Differences within and between FP and AP groups from Screening to Year 3 were studied and considered in relation to our prior cross-sectional study^7^. Within groups, more metabolites were nominally different in the FP group than the AP group. Of note, many of the metabolites that decreased in the FP group had higher concentrations at Screening than AP but ended up being lower than AP by Year 3. Most of the metabolites that changed in the FP group remained relatively stable in the AP group. It is not clear why Screening concentrations of many diverse classes of metabolites should be higher in the FP group; some of these may be compensatory and advantageous, but an absence of stability over time appears to correlate to accelerated functional decline. The majority of the metabolites that decreased in FP were lipid based, including PCs, LPCs, SMs and CEs. This decrease over the 3-year period may reflect faster general degeneration and less available substrate with disease progression, possibly impaired synthesis with progressive pathology, or a preferential shift to catabolism and consumption in the setting of failing oxidative phosphorylation. FP may experience an especially aggressive catabolic process with greater depletion over time; our previous cross-sectional study suggests increasing reliance on FA oxidation with progressive functional decline^7^.

Several of the metabolomic changes suggest increased inflammation and oxidative stress in both FP and AP, with more pronounced changes in FP. Higher levels of Trp and Kyn at Screening in FP may reflect an increasingly inflammatory environment. Excessive catabolism would be expected to result in increasing Kyn; however, the decline observed in Year 3 in FP may result from conversion to downstream products including the neurotoxic metabolite quinolinic acid, which has been reported to be elevated in the striatum and cortex in HD^11,12^. In AP, IPA, an anti-inflammatory metabolite generated from the metabolism of Trp by *Clostridium Sporogenes* in the gut^13^, was higher at Screening but declined by Year 3. This may represent more effective initial compensation in AP, but with time, reduction in Trp levels, and possibly a change in the HD microbiome, it abates. DGs are second messengers that impact signal transduction in numerous pathways, including TOR and S6K signaling via lipase activity, and that protect against of oxidative stress^14^; as a class, they had the most increases with time in AP. This may reflect an advantageous adaptation to counter increasing oxidative damage. The relative stability of Cys in AP compared to the decline in FP is consistent with decreased oxidative stress in AP because Cys is integral for glutathione production^15^. The production of Cys may be more impaired in FP, consistent with reduced cystathionine gamma-lyase (CSE) levels in HD secondary to diminished activating transcription factor 4 (ATF4), a component of the stress response^16^. Lower Cys can also result in the reduction of hydrogen sulfide and coenzyme A^17,18^, the latter of which has implications for the citric acid cycle and FA synthesis. This may explain the greater reductions in FA observed in the FP group at Year 3 compared to AP. Curiously, cystine, the oxidized form of Cys, increased from Screening to Year 3 in AP to a level comparable to FP at Screening; this may reflect extracellular accumulation due to gradual failure of the cystine transport system, known to malfunction in HD^19^. With FP, more prominent cysteine synthetic failure may help explain greater reductions in cystine levels compared to AP.

Ser and Gly were higher in FP at Screening and decline by Year 3, while remaining relatively stable in AP. Gly and D-Ser are co-agonists for the NMDA receptor at synapses and extra synaptic sites, respectively^20^. Hyperactive NMDA receptors are demonstrable in several genetic HD mouse models, displaying aberrant NMDAR-mediated plasticity before the appearance of motor deficits^21–24^. Similarly, transgenic mice expressing mutant full-length human huntingtin show increased NMDAR activity in medium spiny neurons (MSN) extrasynaptically, suggesting a role in toxicity that is contingent on this regional quality^25^. Higher concentrations of Gly may potentiate NMDA-induced GABA release in the striatum, in turn lessening striatal dopamine release^26^. GABA is known to be lower in the striatum of postmortem HD brains^27^ and lower GABA could particularly relate to loss of inhibitory MSN populations in the striatum^28^. GABA concentrations were similar in FP and AP at Screening, with both groups declining by Year 3 (FP nominally significant). How these observations relate to one another in the highly dysregulated HD functional circuitry of the HD striatum is not specifically clear, but higher Gly concentrations and/or lower GABA likely impart some effect on motor phenotype and influence the relative amounts of chorea or dystonia seen with progression. Considering that higher Gly levels correlate with faster functional decline, revisiting Gly or NMDA antagonism as a therapeutic strategy for functional progression in HD may be warranted.

Several metabolites that changed between and within FP and AP were involved in one-carbon metabolism including Ser, Gly, SMs, PCs and Cys. Several PC concentrations were higher at Screening in FP, although PCs declined between Screening and Year 3 in both groups. VLC (> C22) Cer, HexCer and SMs were also higher at Screening in FP and had decreased by Year 3 while remaining relatively stable in AP. This suggests increased inflammation in FP at Screening, consistent with disruption to ceramide sphingolipid pathways and changes to the lipid profile of neuronal membranes, synapses and possibly myelin maturity^29^. Although our data is from plasma, postmortem HD caudate tissue demonstrates decreased abundance of VLC SMs and HexCer^29^. It is notable that wild-type huntingtin interacts with numerous intracellular surfaces including lipid vesicles for transport, mitochondrial membranes, and endoplasmic reticulum^30^. The lipid composition of membranes is suspected to contribute to HD pathogenesis but its role is complex; for example, the addition of SM or ganglioside to lipid monolayers reduced insertion of synthetic mutant huntingtin peptide, but increased vesicle susceptibility to htt-induced permeability^31^. Other than their role as membrane constituents, sphingolipids are induced by stress, so upregulation may serve a compensatory purpose; higher levels of SMs at Screening and clearly steeper decline with FP support recruitment of a more robust compensation that may become exhausted in more impaired individuals^30^. Alternatively, our observations may indicate transient upregulation followed by more severely impaired synthesis in FP. Augmenting sphingolipids and targeting sphingosine-1-phosphate (S1P) may be a potential therapeutic strategy to promote maintenance of function, as de novo sphingolipid synthesis and S1P are reduced in R6/2 HD mice^32^, and S1P receptor agonists have been shown to reduce huntingtin aggregation, improve motor function, and promote survival in R6/2 mice^33^. S1P lyase, the catabolic enzyme for S1P, is upregulated in human HD cortex^34^.

Other metabolomic changes suggested potential alterations in the nitric oxide (NO) (Arg, Cit, GABR) and urea cycles (Arg, Orn and Cit). Arg, a metabolite we previously observed increasing with worsening functional status^9^ and elsewhere reported higher in HD transgenic sheep^35^, was clearly elevated in FP. The administration of Arg accelerates disease progression in HD mice as measured by weight loss, abnormal clasp, and general motor dysfunction, whereas diets absent in Arg slow weight loss^36^. Accumulating Arg suggests alterations in NO synthase (NOS) activity may be part of accelerated functional decline; NO is thought critical for normal striatal function via regulation of dopaminergic neuron sensitivity to cortical and striatal input^37^. Reduced neuronal NOS expression and activity have been observed in R6/1 and symptomatic R6/2 mice, and neuronal NOS has been reported as reduced in the striatum of HD patients^38,39^. While Arg may be accumulating in part from NOS dysfunction, potential shunting effects into the urea cycle pathway also appear prominent, including elevated Cit, Orn, and lower Arg/Orn ratio at Screening in FP. Increased Cit and Orn suggest higher throughput via arginase and as a result elevated circulating levels of urea, a finding previously observed in transgenic sheep models and human HD brain that is thought to be highly neurotoxic^40,41^.

Our data suggest aberrant urea metabolism is a significant factor driving functional decline in HD. Altered urea cycle metabolism may be exacerbated in FP by higher levels of Gln and Orn, resulting in higher Cit levels. Gln elevation in the periphery may be a result of catabolic conditions and muscle wasting, a well-described feature of HD^2^. Its conversion product, Glu, was abnormally elevated at Screening in both FP and AP and declined with time; curiously, the mean concentration was higher in AP, which may be due to variation or reflect a difference in some peripheral pathological process. It is notable that Glu excitotoxicity has been reported for non-neuronal tissues such as bone and the pancreas, a process that has not been well explored in HD^42^. If the intraparenchymal-blood Glu gradient is disrupted in HD, a process whereby plasma Glu is normally significantly higher than in brain intercellular fluids and involves Na+-dependent efflux into the blood, the elevated plasma values we observed may also have implications for brain dysfunction and exacerbate the decreased Glu efflux ratio observed for HD cellular models^42^. Glu has long been suspected to play an excitotoxic role in HD pathogenesis, though precisely how is not understood. Astrocytic reuptake—which accounts for substantially more Glu clearance compared to nerve terminals—appears to be normal or enhanced in R6/2 models, suggesting failed reuptake may not explain any excitotoxicity in the brain^43^. Elevated striatal Glu may underlie the hyperkinetic phenotype in HD through overactivation of direct pathway MSN and selective degeneration of more vulnerable indirect pathway neurons^44^. With progression, declining levels of cortico-striatal glutaminergic input eventually induce direct pathway dysfunction, correlating with increasingly prominent bradykinesia and parkinsonism. Taken together, our data indicate metabolites in the urea and NO cycles appear to vary according to rate of functional progression, suggesting they may present worthwhile therapeutic targets. A diet relatively low in arginine might theoretically lessen the apparent increase in urea cycle output and toxicity, though it remains to be seen if this is feasible or effective in an HD population.

Beyond signifying dysfunction of urea and NO cycle metabolism, elevated Arg and the polyamine pathway may critically influence one another in HD, as polyamines play a role in homeostasis and prevent apoptosis and inflammation^45^. Notably, pathologically expanded polyglutamine proteins (polyQ57) increase expression and activity of arginase in a CAD neuronal cell line, shifting Arg towards the polyamine pathway (via increased Orn production) and away from NOS^46^. PolyQ proteins in CAD neurons are associated with increased protein aggregation and decreased intracellular levels of spermine, which may be a contributing factor for accelerated functional progression^46^. Putrescine and spermine levels were substantially higher at Screening in AP and remained relatively stable in both groups by Year 3. Not only were putrescine levels in FP lower at Screening than AP, but also lower than reported in healthy humans^47^. A polyamine stress response has been described in which ornithine decarboxylase (ODC) activation promotes initial increases in putrescine, spermine and spermidine followed by longer-term decreases, thought to result from a greater enzymatic breakdown of polyamines in comparison to rates of synthesis^48^. A greater stress response is more likely present in FP with more extensive damage, or mutant huntingtin aggregation may be more pronounced in FP and result in diminished intracellular polyamine levels. Alternatively, ODC may have reduced activity in FP leading to decreased putrescine, spermine and spermidine levels. The substantial increase in FP at Screening of Orn and the > twofold change in the putrescine/Orn ratio is consistent with this hypothesis. Polyamines may have therapeutic importance in HD. Spermine reversed object recognition deficits in an animal model of HD via NMDA receptors^49^, while spermine and spermidine increased mutant huntingtin aggregation and cell viability in HEK293 cells expressing mutant or normal huntingtin transfected with NMDA receptor heterodimers^50^. In theory, putrescine-enriched foods may serve a therapeutic purpose given their importance to homeostasis and correlation to faster progression, but more work is needed to understand polyamine metabolism in vivo as it relates to mutant huntingtin. Further prospective study will help clarify biogenic amine roles in the rate of HD progression and potential for treatment.

Glucose levels were higher at Screening in the FP group and remained higher than AP at Year 3. Increased circulating glucose levels in FP are consistent with impaired mitochondrial oxidation and reliance on glycolysis for energetic needs; brain and skeletal muscle mitochondrial dysfunction have previously been described, and progressive myopathy in HD may involve reduced glucose uptake and utilization in skeletal muscle^51,52^. The persistently elevated circulating glucose levels would be expected to contribute to toxicity over time via increased glycation reactions, protein aggregation, and proteostasis imbalance^53^. Glucose intolerance has been speculated as part of HD progression based on early studies and pre-clinical models, though this remains inconclusive^53^. Altered glucose metabolism has been targeted as a therapeutic strategy in HD; the hypoglycemic agent exendin-4, a GLP-1R agonist, increased insulin secretion/sensitivity, motor coordination and lifespan in a HD mouse model^54^. Our data suggest that the FP group may be more prone to glucose intolerance; indeed, one participant was already insulin-dependent at Screening and remained so at Year 3. Fasting was not required for 2CARE blood draws, so values could have been influenced by variability in oral intake, risk factors unrelated to HD, and potentially differences in antipsychotic use; antipsychotics, which promote glucose intolerance, were more commonly used in the FP group at Screening (29% vs 15%) and Year 3 (57% vs. 23%). It is not clear if elevated plasma glucose levels are a consequence of more severe HD or if functional decline in HD is more rapid secondary to unrelated alterations in glucose metabolism, but greater surveillance for glucose intolerance and treatment may be worth testing for a role in lessening functional decline.

Several of the metabolomic changes observed in FP are consistent with a decline in activation of AMPK signaling, a major energy sensor that maintains cellular energy homeostasis^55^. A decrease in NOS activity would lessen SIRT1 activation, and as a result decrease phosphorylation of AMPK. The decline in phosphorylated AMPK could promote insulin resistance and increase circulating glucose levels, as AMPK phosphorylation enhances glucose uptake by muscle cells^55,56^. Spermidine, which was lower at Screening in FP, can also reduce inflammation by activating AMPK signaling^57^. AMPK activation has been shown to increase ODC translation via phosphorylation of the zinc finger protein CNBP in granule cell precursors^58^; this may increase polyamine synthesis, decreasing Orn and Arg in the process, and thereby potentially decrease urea production and toxicity. Reports on AMPK activation in HD have been mixed, with conflicting studies suggesting that AMPK activation in HD could be detrimental by promoting neurodegeneration^6^ or beneficial as a compensatory response protecting dysfunctional and vulnerable neurons in pre-clinical models^59,60^. Our data indirectly implicate AMPK as potentially relevant for functional decline and suggest that AMPK activation may warrant continued exploration as a therapeutic target.

This dataset has several limitations. Only plasma was analyzed, and so while the data can be reasonably considered as reflecting general physiology, our findings may not reflect actual intraneuronal or intraparenchymal brain conditions. AP and FP groups were asymmetric and included small numbers of participants, particularly the fast group; by Year 3, only 3 FP participants were available for analysis. This is perhaps to be expected, with faster disease progression precluding more prolonged trial participation and collection of plasma. Given the number of metabolites of interest, adjustments for multiplicity of statistical tests were not made, and so all p-values are nominal. These data are intended to be hypothesis-generating. We note that several APs already had functional deficits at Screening (those with TFC scores less than 13) that went on to remain stable across the 3-year period, whereas others displayed no functional deficit through time (TFC scores remained 13 from screening onward). It is possible these subgroups are different metabolically, and this may be a source of variation. The TFC Score assays items that are not HD-specific (occupation, finances, chores, activities of daily living, care requirements) but are universally affected with time in HD. Early functional decline commonly includes changes in occupational status and finances, after which other domains (activities of daily living, domestic chores, care level) may not be as sensitive to change in some participants and result in temporary stability after initial change^61^. It is possible other unknown factors or comorbid medical conditions influenced functional rating of these participants at Screening, but for some reason did not exert the same influence through time; nonetheless, they remained stable from screening onward and in aggregate appear to show metabolomic differences compared to those who progress steadily across timepoints. In small datasets, functional decline in HD may not be uniform through time and appear stepwise for some; this contrasts with observations from larger datasets in the literature, which indicate a more monotonic decline^62,63^. Participants were prescribed a number of medications (anxiolytics, antidepressants, antipsychotics) that could possibly influence metabolic markers, though to what extent these effects would be visible in plasma samples is not clear.

Overall, our data suggest greater urea and NO cycle dysfunction correlates to faster disease progression, possibly in part through greater accumulation of urea in the CNS. A notable reduction in the polyamine putrescine is also associated with faster progression; in combination with accumulating ornithine, these data suggest ODC dysfunction may be present in HD. These preliminary data suggest a biomarker panel of only a few metabolites may help predict rate of functional decline, which may have utility in selection of participants for clinical trials. Regarding therapeutic implications of these data, it is intriguing to consider putrescine supplementation for HD patients as a way to bypass low levels associated with faster progression, perhaps accompanied by low dietary arginine and treatment with hypoglycemic agents. It is not clear how to optimize speculative supplementation or deprivation to maintain general health and avoid potential toxicity; these findings will need replication in larger datasets and formal assessment in humans for safety and tolerability, but targeting these pathways synergistically may improve the rate of decline in the progression of HD. Whether an AMPK activator could result in improvement of arginine and glucose levels may also warrant further study.

## Methods

2CARE (ClinicalTrials.gov identifier number NCT00608881 posted on February 6, 2008) was approved by the institutional review boards at 48 participating sites in the United States, Canada, and Australia. The trial was conducted in accordance with the Declaration of Helsinki and International Conference on Harmonization Good Clinical Practice Guidelines. All participants provided written informed consent for their trial participation. A subset of participants provided additional informed consent allowing the use of plasma collected from them during the trial for future research. From this subset of participants, we selected samples for the present analysis. The Institutional Review Board at Cooper University Hospital did not require additional review of research involving deidentified samples from the selected participants, who already provided consent.

2CARE was an HD interventional clinical trial that enrolled 609 participants at 48 sites in North America from 2008 to 2012 and randomized them to either placebo or supplementation with Coenzyme Q10 (2400 mg/day)^8^. The Unified Huntington’s Disease Rating Scale, the clincal rating scale used in the study, is comprised of five domains for assessment: Motor, Cognitive, Behavioral, Independence Scale, and Total Functional Capacity^64^. The Total Functional Capacity (TFC) Score, ranging from 13 (full functional capacity) to 0 (completely incapacitated), assesses five domains: occupation, finances, domestic chores, activities of daily living, and care level. TFC scores define stages of the disease: HD1 (TFC 11–13), HD2 (TFC 7–10), HD3 (TFC 3–6), HD4 (1–2), and HD5 (0). TFC was chosen to explore in relation to the metabolome for this study given its clear relation to disability in daily living and its synthetic quality for capturing motor, behavioral, and cognitive elements of the disease.

The minimum TFC value for participation at Screening was 9, with no restriction on maximum value (i.e., values of 13 were permitted). Plasma collected as part of the study was stored after study completion at − 80 °C. From among these samples, individuals matched at baseline but with different rates of disease progression who received placebo were selected for analysis. To define “fast” and “slow” progression, a slope analysis was conducted from Baseline to Year 3 for change in Total Functional Capacity Score. Participants from the upper 10% (“fast”) and lower 10% (“absent”) for whom plasma was available in sufficient quantities were selected for further analysis. TFC values for these participants across different timepoints are shown in Table 1. For some participants, no progression from Screening was evident by Year 3 (Table 1).

Metabolites shown in Results were selected from participants with fast progression between Screening/Year 0 (7 participants) and Year 3 (3 participants) and AP between Screening/Year 0 (13 participants) and Year 3 (10 participants).

### Sample acquisition and processing

Plasma samples were obtained from the University of Rochester (Rochester NY, USA) and the BioSEND repository (Indiana, USA). Metabolites were extracted from plasma and concentrations obtained using the MxP 500 (Biocrates Life Science AG, Austria) following the manufacturer’s protocol. Metabolites were measured using a Nexera HPLC system (Shimadzu) coupled to a 6500 QTRAP® mass spectrometer (AB Sciex) with an electrospray ionization source. Lipids, acyl carnitines, and hexoses were measured over two injections by flow injection analysis-tandem mass spectrometry (FIA-MS/MS) in positive ionization modes. Amino acids, amino acid related, carboxylic acids, fatty acids, indole derivatives, biogenic amines, bile acids, cresols, alkaloids, amine oxides, hormones, vitamins and cofactors, and nucleobases related metabolites were measured by liquid chromatography-tandem mass spectrometry (LC–MS/MS) from two injections in positive and negative ionization modes. Data from all injections were imported and quantified in Biocrates MetIDQ™ software. Analytes in the LC–MS/MS part were quantified using either external 7-point calibration curves with labeled standards or internally with labeled standards. Analytes in the FIA-MS/MS part were quantified using internal standards^65^. Shorthand notation ^10^ for the lipids are provided in Table S2. 

### Statistical analysis

Descriptive statistics (minimums, maximums, means, standard deviations) were calculated and reported. General estimating equations models were used to account for longitudinal data at all time points and to examine trends in the data. We concluded with running paired t-tests to compare the mean values of the metabolites between Screening and Year 3. p ≤ 0.05 was the significance level; given the exploratory nature of the study, no adjustments for multiplicity were made. All p-values reported are nominal. SAS (SAS Institute, Cary, NC) and SPSS 27 (IBM, Armonk, NY) were used for analysis.

Metabolites with nominally significant p-values for timepoint comparisons were further tabulated. Items of interest included metabolites different from Screening to Year 3 within the fast and absent progression groups, those different at Screening between fast and absent progression groups, those different at Year 3 between fast and absent progression groups, those with differences at both Screening and Year 3 in both fast and absent progression groups, and the magnitude/direction of the changes associated with these differences.

### Supplementary Information

Supplementary Table S1.

Supplementary Table S2.

## Data Availability

The datasets used and/or analyzed during the current study are available from the corresponding authors on request.
