# Supplementary material for: An exploratory metabolomic comparison of participants with fast or absent functional progression from 2CARE, a randomized, double-blind clinical trial in Huntington’s disease
Source: Sci Rep. 2024 Jan 11;14:1101. doi: 10.1038/s41598-023-50553-y (PMC10784537; doi:10.1038/s41598-023-50553-y)
Supplement: Supplementary file 1 — Supplementary Table S1. [file 41598_2023_50553_MOESM1_ESM.docx]

**Table S1.** Average circulating concentrations (µM) of metabolites that were nominally different (p<0.05) or trending (0.05<p<0.10) in plasma between Screening and Year 3 in either Absent Progressors or Fast Progressors or both.

|  | **Absent** | | | **Fast** | | |
| --- | --- | --- | --- | --- | --- | --- |
| **Metabolite** | **Year 0** | **Year 3** | **p-value** | **Year 0** | **Year 3** | **p-value** |
| AconAcid | 9.95 | 7.05 | 0.0106 | 11.86 | 7.79 | 0.0736 |
| C0 | 34.86 | 35.19 |  | 40.3 | 34.77 | 0.075 |
| C10 | 0.22 | 0.28 | 0.0568 | 0.24 | 0.16 | 0.0696 |
| C141 | 0.04 | 0.06 |  | 0.06 | 0.02 | 0.0329 |
| C16 | 0.1 | 0.11 |  | 0.11 | 0.07 | 0.0044 |
| C18 | 0.05 | 0.05 |  | 0.05 | 0.03 | 0.0024 |
| C181 | 0.08 | 0.09 |  | 0.09 | 0.06 | 0.0115 |
| C182 | 0.04 | 0.05 |  | 0.05 | 0.04 | 0.062 |
| CE141 | 0.05 | 0.12 |  | 0.21 | 0 | 0.0127 |
| CE150 | 8.53 | 8.5 |  | 11.73 | 6.15 | 0.0788 |
| CE160 | 249.86 | 256.1 |  | 341.5 | 190.67 | 0.0012 |
| CE170 | 7.21 | 6.9 |  | 11.41 | 5.44 | 0.0485 |
| CE171 | 3.64 | 3.25 |  | 5.79 | 0.86 | 0.0498 |
| CE180 | 19.08 | 19.91 |  | 27.9 | 14.83 | 0.001 |
| CE181 | 446.79 | 438.9 |  | 668.5 | 338.33 | 0.0034 |
| CE182 | 1762.36 | 1731.7 |  | 2249.25 | 1404.33 | 0.0036 |
| CE183 | 87.54 | 80.16 |  | 122.81 | 83.3 | 0.0313 |
| CE204 | 292.07 | 309.5 |  | 436.75 | 246 | 0.0033 |
| CE222 | 0.8 | 0.66 |  | 1.79 | 0.41 | 0.0684 |
| CE225 | 3.78 | 3.28 |  | 4.72 | 2.89 | 0.0228 |
| Cer d16:1/23:0 | 0.09 | 0.12 |  | 0.14 | 0 | 0.0026 |
| Cer d16:1/24:0 | 0.17 | 0.19 |  | 0.21 | 0.14 | 0.0469 |
| Cer d18:0/22:0 | 0.13 | 0.12 |  | 0.15 | 0.02 | 0.0496 |
| Cer d18:1/14:0 | 0.05 | 0.03 |  | 0.07 | 0.01 | 0.0073 |
| Cer d18:1/16:0 | 0.43 | 0.42 |  | 0.45 | 0.31 | 0.0012 |
| Cer d18:1/22:0 | 0.97 | 1.01 |  | 1.07 | 0.67 | 0.001 |
| Cer d18:1/23:0 | 0.91 | 0.96 |  | 1.13 | 0.67 | 0.0005 |
| Cer d18:1/24:0 | 2.68 | 2.37 |  | 2.91 | 1.84 | 0.0254 |
| Cer d18:1/24:1 | 1.07 | 1.15 |  | 1.17 | 0.82 | 0.0066 |
| Cer d18:1/25:0 | 0.33 | 0.3 |  | 0.37 | 0.24 | 0.0014 |
| Cer d18:2/22:0 | 0.28 | 0.28 |  | 0.31 | 0.2 | 0.01 |
| Cer d18:2/24:0 | 0.51 | 0.51 |  | 0.58 | 0.38 | 0.0045 |
| Cer d18:2/24:1 | 0.23 | 0.25 |  | 0.27 | 0.16 | <.0001 |
| Cit | 34.66 | 31.71 |  | 45.89 | 33.17 | 0.0081 |
| Cys | 51.62 | 52.34 |  | 55.03 | 42 | 0.0297 |
| Cystine | 33.66 | 40.13 | 0.0346 | 42.05 | 39.3 |  |
| DG140_14 0 | 0.04 | 0.04 |  | 0.04 | 0.03 | 0.0147 |
| DG160_18 2 | 2.09 | 3.27 | 0.0469 | 2.88 | 1.84 |  |
| DG170_18 1 | 0.51 | 0.38 | 0.0977 | 0.65 | 0.21 | 0.003 |
| DG181_18 1 | 3.39 | 4.99 | 0.0281 | 4.98 | 2.15 | 0.0597 |
| DG181_18 2 | 7.05 | 11.8 | 0.0052 | 9.2 | 6.42 |  |
| DG181_18 3 | 0.21 | 0.45 | 0.0206 | 0.32 | 0.21 |  |
| DG182_18 2 | 4.32 | 8.34 | 0.0028 | 6.02 | 6.82 |  |
| EPA | 0.75 | 0.89 |  | 1.41 | 0.16 | 0.0241 |
| FA181 | 139.08 | 168.63 |  | 132.9 | 49.47 | 0.0869 |
| GABA | 0.2 | 0.16 | 0.0587 | 0.19 | 0.15 | 0.0362 |
| GCA | 0.21 | 0.27 | 0.0652 | 0.65 | 0.42 |  |
| GCDCA | 0.83 | 1.16 | 0.0381 | 2.3 | 1.41 |  |
| Gln | 541.12 | 631.4 |  | 778.29 | 649 | 0.0669 |
| Glu | 143.26 | 48.35 | 0.0024 | 101.69 | 39.67 |  |
| Gly | 277.43 | 286.9 |  | 404.57 | 322.33 | 0.0067 |
| Hex2Cerd 181160 | 2.62 | 2.59 |  | 2.69 | 1.65 | 0.0079 |
| Hex2Cerd 181180 | 0.15 | 0.16 |  | 0.17 | 0.13 | 0.0466 |
| Hex2Cerd 181240 | 0.22 | 0.17 | 0.0277 | 0.2 | 0.14 |  |
| Hex2Cerd 181241 | 0.39 | 0.37 |  | 0.37 | 0.26 | 0.0758 |
| Hex3Cerd 181241 | 0.34 | 0.37 |  | 0.35 | 0.27 | 0.0906 |
| HexCerd1 81230 | 1.31 | 1.37 |  | 1.65 | 1.34 | 0.0753 |
| HexCerd1 81240 | 1.64 | 1.86 |  | 2.05 | 1.6 | 0.0037 |
| HexCerd1 81241 | 3.22 | 3.46 |  | 3.7 | 3.04 | 0.0408 |
| HexCerd182220 | 0.48 | 0.52 |  | 0.61 | 0.17 | 0.0018 |
| His | 85.4 | 79.52 |  | 107.1 | 92.37 | 0.0864 |
| IAA3 | 2.79 | 2.38 | 0.0823 | 5.26 | 1.64 | 0.0003 |
| IPA3 | 0.94 | 0.72 | 0.0484 | 0.87 | 0.62 |  |
| Kynurenine | 1.97 | 1.87 |  | 2.14 | 1.53 | 0.0008 |
| lysoPCaC160 | 106.89 | 77.23 | 0.0134 | 163.49 | 63.73 | 0.0008 |
| lysoPCaC161 | 3.29 | 2.31 | 0.0198 | 4.4 | 1.69 | 0.0065 |
| lysoPCaC170 | 2.01 | 1.47 | 0.0029 | 3.26 | 1.27 | 0.005 |
| lysoPCaC180 | 30.9 | 23.19 | 0.0129 | 52.21 | 19.6 | 0.0004 |
| lysoPCaC181 | 22.04 | 17.34 | 0.0184 | 34.7 | 14.4 | <.0001 |
| lysoPCaC182 | 34.91 | 31.61 |  | 55.96 | 35.37 | 0.004 |
| lysoPCaC203 | 2.38 | 2.09 |  | 3.17 | 2.01 | 0.0191 |
| lysoPCaC204 | 6.78 | 6.14 |  | 10.99 | 5.72 | 0.0004 |
| lysoPCaC240 | 0.22 | 0.2 |  | 0.25 | 0.19 | 0.0179 |
| lysoPCaC261 | 0.14 | 0.14 |  | 0.16 | 0.13 | 0.0777 |
| lysoPCaC281 | 0.31 | 0.31 |  | 0.39 | 0.27 | 0.0209 |
| MetSO | 0.87 | 0.54 | 0.0762 | 0.77 | 1.19 |  |
| Orn | 55.09 | 51.81 |  | 93.13 | 67.2 | 0.0502 |
| PCaaC281 | 3.76 | 3.66 |  | 4.15 | 2.9 | 0.0011 |
| PCaaC320 | 16.29 | 16.31 |  | 16.35 | 11.83 | 0.0123 |
| PCaaC323 | 0.85 | 0.77 |  | 0.87 | 0.56 | 0.0288 |
| PCaaC341 | 224.57 | 214.1 |  | 235.5 | 147.1 | 0.0023 |
| PCaaC342 | 438.64 | 441.1 |  | 454.75 | 351.33 | 0.0014 |
| PCaaC343 | 22.37 | 21.01 |  | 21.74 | 17 | 0.0674 |
| PCaaC360 | 1.83 | 1.95 |  | 1.89 | 1.27 | 0.0554 |
| PCaaC361 | 57.18 | 51.23 |  | 63.73 | 37.53 | 0.0131 |
| PCaaC362 | 269.79 | 262.4 |  | 299.5 | 215.33 | 0.0059 |
| PCaaC363 | 162.82 | 149.39 |  | 172.5 | 127.67 | 0.0057 |
| PCaaC364 | 213.86 | 204.2 |  | 246.75 | 184 | 0.0117 |
| PCaaC380 | 3.03 | 3.18 |  | 3.21 | 2.2 | 0.0558 |
| PCaaC384 | 118.6 | 115.67 |  | 144.98 | 104.83 | 0.0561 |
| PCaaC385 | 64.91 | 64.09 |  | 78.95 | 52.63 | 0.014 |
| PCaaC402 | 0.34 | 0.29 | 0.0139 | 0.46 | 0.22 | <.0001 |
| PCaaC403 | 0.59 | 0.52 |  | 0.68 | 0.38 | 0.0008 |
| PCaaC404 | 3.83 | 3.32 |  | 4.37 | 2.94 | 0.037 |
| PCaaC421 | 0.35 | 0.32 |  | 0.36 | 0.25 | 0.0228 |
| PCaaC422 | 0.28 | 0.26 |  | 0.32 | 0.22 | 0.0074 |
| PCaaC424 | 0.22 | 0.19 |  | 0.27 | 0.16 | 0.0007 |
| PCaaC425 | 0.38 | 0.32 | 0.0944 | 0.38 | 0.23 | 0.0018 |
| PCaaC426 | 0.47 | 0.43 |  | 0.51 | 0.33 | 0.0074 |
| PCaeC301 | 0.16 | 0.17 |  | 0.18 | 0.11 | 0.0655 |
| PCaeC302 | 0.1 | 0.09 |  | 0.12 | 0.08 | 0.0521 |
| PCaeC321 | 3.08 | 3.16 |  | 3.41 | 2.43 | 0.0015 |
| PCaeC322 | 0.84 | 0.81 |  | 0.89 | 0.62 | 0.0016 |
| PCaeC340 | 1.81 | 1.76 |  | 1.91 | 1.17 | 0.0487 |
| PCaeC341 | 11.76 | 11.05 |  | 12.59 | 8.28 | 0.0051 |
| PCaeC342 | 14.24 | 13.66 |  | 15.25 | 10.9 | 0.0082 |
| PCaeC343 | 11.28 | 10.03 |  | 12.09 | 8.41 | 0.0018 |
| PCaeC361 | 8.85 | 8.18 |  | 10.37 | 6.42 | 0.005 |
| PCaeC362 | 17.16 | 16.83 |  | 18.6 | 14 | 0.0181 |
| PCaeC363 | 10.38 | 9.25 |  | 11.5 | 7.87 | 0.0071 |
| PCaeC364 | 20.74 | 19.63 |  | 26.58 | 17.03 | 0.0034 |
| PCaeC365 | 14.27 | 14.01 |  | 17.14 | 11.62 | 0.0027 |
| PCaeC381 | 0.3 | 0.2 |  | 0.78 | 0.2 | 0.0214 |
| PCaeC382 | 2.23 | 2.09 |  | 2.9 | 1.77 | 0.0109 |
| PCaeC383 | 5 | 4.28 |  | 6.46 | 3.87 | 0.0287 |
| PCaeC384 | 15.37 | 14.38 |  | 18.7 | 12.43 | 0.0072 |
| PCaeC385 | 20.71 | 20.2 |  | 26.45 | 17.3 | 0.0006 |
| PCaeC386 | 8.5 | 8.84 |  | 9.82 | 6.54 | 0.0098 |
| PCaeC401 | 1.41 | 1.33 |  | 1.6 | 1.1 | 0.0042 |
| PCaeC402 | 1.72 | 1.68 |  | 2.07 | 1.26 | 0.0006 |
| PCaeC403 | 1.27 | 1.1 |  | 2.13 | 0.84 | <.0001 |
| PCaeC404 | 2.88 | 2.54 | 0.0928 | 3.69 | 2.09 | 0.0005 |
| PCaeC405 | 4.1 | 3.68 |  | 5.1 | 3.05 | 0.004 |
| PCaeC406 | 4.88 | 4.94 |  | 5.2 | 3.43 | 0.0247 |
| PCaeC421 | 0.47 | 0.43 |  | 0.53 | 0.36 | 0.0024 |
| PCaeC422 | 0.59 | 0.54 |  | 0.7 | 0.43 | 0.0001 |
| PCaeC423 | 0.89 | 0.81 | 0.0874 | 0.96 | 0.6 | 0.0003 |
| PCaeC424 | 1.13 | 0.98 | 0.0845 | 1.23 | 0.72 | 0.0067 |
| PCaeC425 | 2.31 | 2.13 |  | 2.52 | 1.65 | 0.0055 |
| PCaeC443 | 0.19 | 0.17 |  | 0.2 | 0.14 | 0.0128 |
| PCaeC445 | 1.84 | 1.61 |  | 1.72 | 1.19 | 0.016 |
| PCaeC446 | 1.75 | 1.59 |  | 1.68 | 1.14 | 0.0781 |
| Sarcosine | 5.62 | 4.17 | 0.0472 | 5.5 | 4.14 |  |
| SDMA | 0.55 | 0.59 |  | 0.6 | 0.45 | 0.0109 |
| SMC161 | 14.88 | 14.4 |  | 17.11 | 11.84 | <.0001 |
| SMC180 | 19.06 | 19.05 |  | 22.31 | 14.43 | 0.0132 |
| SMC181 | 9.45 | 9.31 |  | 11.2 | 7.06 | 0.0165 |
| SMC202 | 0.38 | 0.36 |  | 0.43 | 0.21 | 0.0519 |
| SMC240 | 17.47 | 16.32 |  | 17.85 | 12.4 | 0.0004 |
| SMC241 | 34.39 | 33.75 |  | 35.8 | 26.67 | 0.0032 |
| SMC261 | 0.28 | 0.27 |  | 0.3 | 0.25 | 0.0798 |
| SMOHC141 | 6.19 | 6.38 |  | 7.75 | 5.5 | 0.0007 |
| SMOHC161 | 3.01 | 3.05 |  | 3.72 | 2.54 | 0.0077 |
| SMOHC221 | 11.38 | 10.79 |  | 12.76 | 8.77 | 0.0005 |
| SMOHC222 | 8.33 | 7.86 |  | 10.06 | 6.75 | <.0001 |
| SMOHC241 | 1.02 | 0.99 |  | 1.13 | 0.95 | 0.0444 |
| t4OHPro | 12.33 | 8.38 | 0.002 | 16.93 | 13.03 |  |
| Trp | 55.11 | 51.81 |  | 66.86 | 49.2 | 0.0064 |
| Tyr | 65.96 | 58.78 |  | 95.21 | 68.33 | 0.0866 |
| Xanthine | 1.8 | 2.3 | 0.0904 | 2.1 | 1.82 |  |
